# Persistent Low-Risk and High-Risk Human Papillomavirus Infections of the Uterine Cervix in HIV-Negative and HIV-Positive Women

**DOI:** 10.3389/fpubh.2017.00178

**Published:** 2017-07-21

**Authors:** Sally N. Adebamowo, Oluwatoyosi Olawande, Ayotunde Famooto, Eileen O. Dareng, Richard Offiong, Clement A. Adebamowo, for the H3Africa ACCME Research Group

**Affiliations:** ^1^Department of Epidemiology and Public Health, University of Maryland School of Medicine, Baltimore, MD, United States; ^2^University of Maryland Comprehensive Cancer Center, University of Maryland School of Medicine, Baltimore, MD, United States; ^3^Center for Bioethics and Research, Ibadan, Nigeria; ^4^Office of Strategic Information and Research, Institute of Human Virology Nigeria, Abuja, Nigeria; ^5^Department of Public Health and Primary Care, University of Cambridge, Cambridge, United Kingdom; ^6^Department of Obstetrics and Gynecology, University of Abuja Teaching Hospital, Abuja, Nigeria; ^7^Institute of Human Virology, University of Maryland School of Medicine, Baltimore, United States; ^8^Institute of Human Virology Nigeria, Abuja, Nigeria

**Keywords:** human papillomavirus, HIV, Nigeria, cervical cancer, prevalence, persistence and multiple infections

## Abstract

**Background:**

The prevalence, persistence, and multiplicity of human papillomavirus (HPV) infection appears different comparing HIV-positive to HIV-negative women. In this study, we examined prevalent, persistent, and multiple low- and high-risk cervical HPV infections in HIV-negative and HIV-positive women.

**Methods:**

We studied 1,020 women involved in a study of HPV infection using SPF_25_/LiPA_10_. Two study visits were scheduled, at enrollment and 6 months afterward. At each study visit, research nurses used a cervical brush to collect samples of exfoliated cervical cells from the cervical os, from all the study participants. Exact logistic regression models were used to estimate associations between HIV and HPV infections.

**Results:**

The mean (SD) age of the study participants was 38 (8) years, 56% were HIV-negative and 44% were HIV-positive. Among HIV-negative women at baseline, single low-risk HPV (lrHPV) infections occurred in 12%; multiple lrHPV in 2%; single high-risk human papillomavirus (hrHPV) infections in 9%, and multiple hrHPV infections in 2%. Single lrHPV infections were persistent in 6%, but there was no persistent multiple lrHPV infections. Single hrHPV infections were persistent in 4% while multiple hrHPV infections were persistent in 0.3%. Among HIV-positive women at baseline, single lrHPV infections occurred in 19%, multiple lrHPV in 6%, single hrHPV infections in 17%, and multiple hrHPV infections occurred in 12%. Single lrHPV infections were persistent in 9%, multiple lrHPV infections in 0.6%, single hrHPV infections in 13%, while multiple hrHPV were persistent in 3%. Prevalent, persistent, and multiple infections were more common in HIV-positive women, compared to HIV-negative women. In multivariate models adjusted for age, marital status, socioeconomic status, age at sexual initiation, and douching, the odds ratios comparing HIV-positive to HIV-negative women, were 2.09 (95% CI 1.47–2.97, *p* < 0.001) for prevalent lrHPV, 1.26 (95% CI 0.66–2.40, *p* 0.47) for persistent lrHPV infections, 3.38 (95% CI 2.34–4.87, *p* < 0.001) for prevalent hrHPV, and 4.49 (95% CI 2.26–8.91, *p* < 0.001) for persistent hrHPV infections.

**Conclusion:**

HIV infection was associated with higher prevalence of lrHPV, hrHPV, and persistence hrHPV infections, but not persistent lrHPV infections.

## Introduction

Persistent high-risk human papillomavirus (hrHPV) infection of the uterine cervix is a necessary cause of cervical cancer. hrHPV infection is highly prevalent with some 80% of women being infected at some point in their lives (1). These infections typically start in early adulthood, shortly after sexual initiation, but their prevalence falls rapidly and the infection persists in only approximately 10% of women older than 30 years of age. Cervical cancer occurs in a small proportion (12%) of women who have persistent hrHPV infections. Most research on hrHPV infections in Sub-Saharan Africa (SSA) to date have focused on reporting prevalent infections whose association with cervical carcinogenesis is weak, rather than persistent infections. This is due to challenges with participants’ follow-up and lack of sustained systematic human papillomavirus (HPV) DNA tests’-based screening programs.

Previous studies suggest that there is greater heterogeneity of HPV types in populations with high prevalence of HPV infections, thereby reducing the relative prevalence of HPV types 16 and 18 (2). The most commonly reported hrHPV types in SSA are HPV types 35, 52, and 58 (3–8). There is a dearth of data on the risk of prevalent and persistent, single, and multiple infections with hrHPV among HIV-negative and -positive women, especially from West Africa.

Low-risk HPV (lrHPV) infections are similarly ubiquitous and are associated with common, flat plane, anogenital warts, and oral lesions (9). These diseases cause significant morbidity, are difficult to treat, and have high health systems’ costs; nevertheless, there are very few studies of lrHPV in SSA (10, 11). Furthermore, little is known about the prevalence and persistence of combined lrHPV and hrHPV infections in SSA or whether interactions between lrHPV and hrHPV infections are associated with persistence of either infections. More research on prevalence, persistence, and multiplicity of HPV infections of the uterine cervix in SSA women is urgently required in order to ascertain the role of different HPV types in cervical cancer in this population.

HIV infection is associated with increased risk of cervical cancer. Several studies have shown that HIV-positive women are more likely to have prevalent and multiple HPV infections, compared to HIV-negative women (3, 4, 8, 12). Some studies show that the type distribution of HPV in HIV-positive women is different from that in HIV-negative women (13, 14) but not all studies support this finding (4). More studies examining the distribution of HPV infections of the uterine cervix by HIV status are warranted. The results of such studies would contribute to understanding HIV-associated cervical cancer and choice of cervical cancer prevention in people living with HIV/AIDS (PLWA).

In this study, we examined the prevalence, persistence, and multiplicity of infections of the uterine cervix by lrHPV and hrHPV types among HIV-negative and HIV-positive women in Nigeria.

## Materials and Methods

### Study Population

We studied 1,020 women who were enrolled in a study of HPV infection and cervical cancer at National Hospital, Abuja and University of Abuja Teaching Hospital, Nigeria, between 2012 and 2014. All study participants were 18 years or older, had history of prior vaginal sex, were not pregnant, and had an intact uterus at enrollment. Interviewers used questionnaires to collect data on sociodemographic characteristics, sexual and reproductive history, and HIV status, which were confirmed from medical records. During the baseline visit and the follow-up visit, which was scheduled to occur 6 months after enrollment, research nurses used a cervical brush to collect samples of exfoliated cervical cells from the cervical os, from all the study participants.

### HPV Detection by SPF_10_/LiPA_25_

We extracted DNA from cervical exfoliated cells as previously described (15). Samples were tested for the presence of HPV DNA by hybridization of SPF_10_ amplimers to a mixture of general HPV probes recognizing a broad range of high-risk, low-risk, and possible hrHPV genotypes in a microtiter plate format, as described previously (16). All samples determined to be HPV DNA positive by SPF_10_ DNA enzyme immunoassay were genotyped using the LiPA_25_ version 1. The LiPA_25_ assay provides type-specific information for 25 different HPV genotypes simultaneously and identifies infection by one or more of 13 hrHPV genotypes: 16, 18, 31, 33, 35, 39, 45, 51, 52, 56, 58, 59, and 68 (17, 18). However, the test does not differentiate between HPV 68 and 73, so we defined this HPV genotype, HPV68/73, as low-risk. The test can identify lrHPV types 6, 11, 34, 40, 42, 43, 44, 53, 54, 66, 70, and 74. The test also identifies unspecified HPV genotypes, HPV U, which we defined as low risk. We defined HPV infection as prevalent if at least one HPV genotype was detected by the SPF_10_/LiPA_25_ test in a sample provided at the baseline visit; and persistent if at least one HPV genotype was detected by the SPF_10_/LiPA_25_ test in each of the samples provided at consecutive baseline and follow-up visits.

### Statistical Analysis

In order to compute socioeconomic status (SES) in a low resource environment where income data is sparse, we generated wealth index data as previously described (19). In summary, we used principal components analysis with varimax rotation to compute factor scores based on the sum of the ownership of household items weighted by their factor loading. We sorted the data on the first principal component, which had the highest eigenvalue and divided all respondents into three categories based on its value. Participants in the lowest 40% were categorized as low SES, the middle 40% were categorized as middle SES, and the top 20% were categorized as high SES. The validity and reproducibility of wealth index has been examined in previous studies and it correlates well with other measures of wealth in environments without reliable expenditure data (19).

We used *t*-tests to assess differences in the distribution of continuous variables between groups and χ^2^ and Fisher’s exact tests for categorical variables. We used exact logistic regression models to evaluate factors associated with HPV infection and between HIV and HPV infections in the study population (20, 21). We conducted age-adjusted analyses of demographic and behavioral characteristics of the participants and their associations with HPV infections. We selected those variables that were associated at *p* < 0.20 for inclusion in multivariate models. All the *p*-values reported were two-sided. All analyses were performed using SAS 9.3 for UNIX statistical software (SAS Institute, Cary, NC, USA).

### Ethics

The study was conducted according to the Nigerian National Code for Health Research Ethics. Ethical approval to conduct this study was obtained from the Institute of Human Virology Nigeria research ethics committee. Written informed consent was obtained from all participants before enrollment in the study, in accordance with the Declaration of Helsinki.

## Results

**Figure 1:**
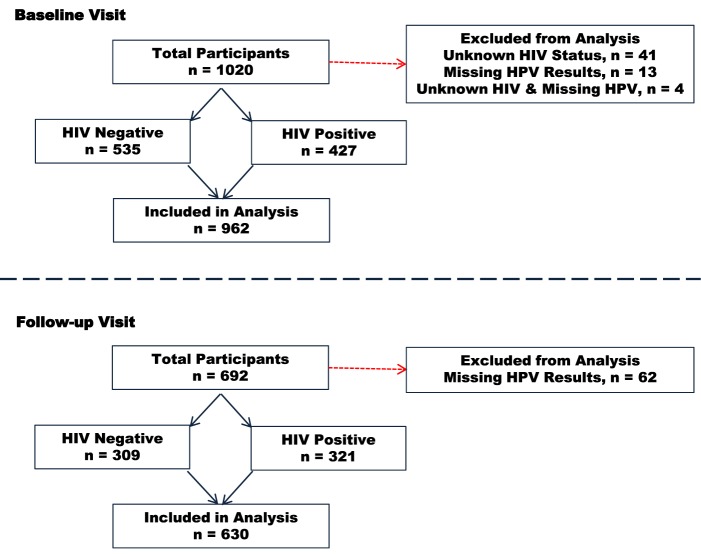
Participant flow chart.

Of the 1,020 participants enrolled at baseline, we excluded 58 participants due to missing results (41 missing HIV results, 13 missing baseline HPV results, and 4 missing both HIV and baseline HPV results) leaving 962 women in the study. Some 72% (692/962) of these women completed follow-up visits. Of these, we excluded 62 participants whose HPV results were missing at follow-up leaving 630 women who had complete data for analysis for persistent HPV infections. The median time interval between the baseline and follow-up visits was 9 months. The participants’ flow through the study is depicted in Figure 1.

### HIV-Negative Women

Most of the women (56%, 535/962) were HIV-negative at baseline. The mean (SD) age of the HIV-negative women was 38 (8) years while their mean (SD) body mass index (BMI) (kilogram per square meter) was 29.0 (8.5). The characteristics of the study participants at baseline are shown in Table 1.

**Table 1:** Baseline characteristics of the study population.

Characteristics	Total *n* = 962	HIV-negative *n* = 535	HIV-positive *n* = 427
		**Mean (SD)**	

Age, years	38 (±8)	38 (8)	37 (7)
Body mass index (BMI), kg/m^2^	28.1 (11.9)	29.0 (8.5)	26.9 (15.2)
Age at sexual initiation, years	20 (±4)	21 (4)	19 (3)
Total sex partners	3 (±3)	3 (2)	4 (4)

		***n* (%)**	

**Age, years**			
18–29	127 (13)	72 (13)	55 (13)
30–39	447 (47)	222 (42)	225 (53)
40–49	317 (33)	196 (37)	121 (28)
≥50	71 (7)	45 (8)	26 (6)
**BMI, kg/m^2^**			
Normal weight, 18.5–24.9	303 (33)	136 (27)	167 (42)
Overweight, 25.0–29.9	324 (36)	184 (36)	240 (35)
Obese, ≥30.0	282 (31)	192 (37)	90 (23)
**Marital status**			
Married	647 (67)	421 (79)	226 (53)
Not married	315 (33)	114 (21)	201 (47)
**Education**			
≤6 years	104 (11)	45 (8)	59 (14)
Secondary	602 (63)	294 (55)	308 (72)
Tertiary	254 (26)	195 (37)	59 (14)
**Socioeconomic status**			
Low	375 (39)	152 (28)	223 (52)
Middle	384 (40)	226 (43)	158 (37)
High	203 (21)	157 (29)	46 (11)
**Age at sexual initiation, years**			
<18	216 (23)	95 (18)	121 (29)
18–20	377 (40)	196 (47)	181 (44)
21–23	180 (19)	113 (21)	67 (16)
>24	174 (18)	127 (24)	47 (11)
**Total sex partners**			
1	249 (26)	179 (33)	70 (16)
2–3	393 (41)	217 (41)	176 (41)
4–5	197 (21)	91 (17)	106 (16)
>5	119 (12)	47 (9)	72 (17)
**Contraceptive use**			
Yes	405 (42)	266 (50)	139 (32)
No	557 (58)	269 (50)	288 (68)
Vaginal pH			
<4.5	73 (8)	47 (9)	26 (6)
4.5–5.5	68 (7)	34 (6)	34 (8)
>5.5	821 (85)	454 (85)	367 (86)
**Menopausal status**			
Premenopausal	813 (85)	443 (84)	370 (87)
Postmenopausal	140 (15)	86 (16)	54 (13)
**Douching**			
Yes	606 (63)	319 (60)	287 (67)
No	355 (37)	216 (40)	139 (33)

Presence of any type of HPV infection of the cervix was detected in 22% (114/535) of these HIV-negative women at baseline, while 10% (31/309) had persistence of any type of HPV infections of the uterine cervix. The infection involved a single HPV type at baseline in 17% (91/535) of these women and there was persistence of any type of HPV infection in 10% (30/309). Multiple infections by any type of HPV were present in 4.3% (23/535) at baseline but only 0.3% (1/309) had persistent multiple HPV infections (Table 2).

**Table 2:** Prevalence of human papillomavirus (HPV) infections in the study population, *n* (%).

Prevalent infections	All *n* = 962	HIV-negative *n* = 535	HIV-positive *n* = 427	*p*-Value
**All prevalent HPV infections**				
Any HPV	300 (31.2)	114 (21.3)	186 (43.5)	<0.001
Low risk	181 (18.8)	72 (13.5)	109 (25.5)	<0.001
High risk	181 (18.8)	57 (10.6)	124 (29.0)	<0.001
**Single prevalent HPV**				
Any HPV	191 (19.6)	91 (17.0)	100 (23.4)	0.01
Low risk	145 (15.1)	63 (11.9)	82 (19.2)	0.001
High risk	119 (12.4)	47 (8.8)	72 (16.9)	<0.001
**Multiple prevalent HPV**				
Any HPV	109 (11.3)	23 (4.3)	86 (20.1)	<0.001
Low risk	36 (3.7)	9 (1.7)	27 (6.3)	<0.001
High risk	62 (6.4)	10 (1.9)	52 (12.2)	<0.001

**Persistent infections**	**All** ***n* = 630**	**HIV-negative** ***n* = 309**	**HIV-positive** ***n* = 321**	

**All persistent HPV infections**				
Any HPV	107 (16.9)	31 (10.0)	76 (23.7)	<0.001
Low risk	50 (7.9)	19 (6.2)	31 (9.7)	0.10
High risk	63 (10.0)	12 (3.9)	51 (15.9)	<0.001
**Single persistent HPV**				
Any HPV	92 (14.6)	30 (9.7)	62 (19.3)	0.001
Low risk	48 (7.6)	19 (6.2)	29 (9.0)	0.18
High risk	53 (8.4)	11 (3.6)	42 (13.1)	<0.001
**Multiple persistent HPV**				
Any HPV	15 (2.4)	1 (0.3)	14 (4.4)	<0.001
Low risk	2 (0.3)	0 (0.0)	2 (0.6)	0.49
High risk	10 (1.6)	1 (0.3)	9 (2.8)	0.02

Single lrHPV infections were present in 12% (63/535) and multiple lrHPV infections were present in 2% (9/535) of the women at baseline, while single hrHPV infections were present in 9% (47/535) of the women and multiple hrHPV infections were present in 2% (10/535) of the women at baseline. Single lrHPV infections were persistent in 6% (19/309), but we did not observe persistent infections with multiple lrHPV types. Single hrHPV infections were persistent in 4% (11/309) of the women and multiple hrHPV infections were persistent in 0.3% (1/309) of the women.

The commonest prevalent lrHPV types detected in these HIV-negative women were unspecified HPV types (31/535, 5.8%), HPV53 (10/535, 1.9%), and HPV68/73 (10/535, 1.9%) while the commonest persistent lrHPV types were unspecified HPV types (7/309, 2.3%) and HPV 68/73 (5/309, 1.6%). The commonest prevalent hrHPV types were HPV52 (16/535, 3.0%) and HPV18 (9/535, 1.7%). HPV16 was detected in only 0.8% (4/535) of these women at baseline (Table 3), while the commonest persistent hrHPV types were HPV52 (5/309, 1.6%) and HPV 35 (3/309, 1.0%). We did not find any persistent infections with HPV16 or HPV18 (Table 4).

**Table 3:** Prevalence and persistence of type-specific low-risk human papillomavirus (HPV) in the total study population and by HIV status, *n* (%).

HPV type	Prevalent HPV				Persistent HPV			
	Total	HIV-negative	HIV-positive	*p*-Value	Total	HIV-negative	HIV-positive	*p*-Value
	
	*n* = 962	*n* = 535	*n* = 427		*n* = 630	*n* = 309	*n* = 321	
HPV 6	10 (1.0)	4 (0.8)	6 (1.4)	0.35	1 (0.2)	0 (0.0)	1 (0.3)	1.00
HPV 11	15 (1.6)	6 (1.1)	9 (2.1)	0.29	2 (0.3)	1 (0.3)	1 (0.3)	1.00
HPV 34	1 (0.1)	0 (0.0)	1 (0.2)	0.44	0 (0.0)	0 (0.0)	0 (0.0)	–
HPV 40	4 (0.4)	1 (0.2)	3 (0.7)	0.32	0 (0.0)	0 (0.0)	0 (0.0)	–
HPV 42	3 (0.3)	0 (0.0)	3 (0.7)	0.08	1 (0.2)	0 (0.0)	1 (0.3)	1.00
HPV 43	6 (0.6)	2 (0.4)	4 (0.9)	0.41	2 (0.3)	0 (0.0)	2 (0.6)	0.49
HPV 44	22 (2.3)	4 (0.8)	18 (4.2)	<0.01	7 (1.1)	0 (0.0)	7 (2.2)	0.01
HPV 53	21 (2.2)	10 (1.9)	11 (2.6)	0.50	4 (0.6)	2 (0.6)	2 (0.6)	1.00
HPV 54	17 (1.8)	3 (0.6)	14 (3.3)	<0.01	3 (0.5)	2 (0.6)	1 (0.3)	0.61
HPV 66	28 (2.9)	5 (0.9)	23 (5.4)	<0.01	9 (1.4)	1 (0.3)	8 (2.5)	0.03
HPV 70	16 (1.7)	4 (0.8)	12 (2.8)	0.01	6 (0.9)	1 (0.3)	5 (1.6)	0.21
HPV 74	11 (1.1)	2 (0.4)	9 (2.1)	0.01	1 (0.2)	0 (0.0)	1 (0.3)	1.00
HPV 68/73	16 (1.8)	10 (2.2)	6 (1.4)	0.62	5 (0.8)	5 (1.6)	0 (0.0)	0.02
Unspecified (U)	54 (5.6)	31 (5.8)	23 (5.4)	0.88	11 (1.7)	7 (2.3)	4 (1.3)	0.37

**Table 4:** Prevalence and persistence of type-specific high-risk human papillomavirus (HPV) in the total study population and by HIV status, *n* (%).

HPV type	Prevalent HPV				Persistent HPV			
	Total	HIV-negative	HIV-positive	*p*-Value	Total	HIV-negative	HIV-positive	*p*-Value
	
	*n* = 962	*n* = 535	*n* = 427		*n* = 630	*n* = 309	*n* = 321	
HPV 16	17 (1.8)	4 (0.8)	13 (3.0)	0.01	4 (0.6)	0 (0.0)	4 (1.3)	0.12
HPV 18	31 (3.2)	9 (1.7)	22 (5.1)	<0.01	4 (0.6)	0 (0.0)	4 (1.3)	0.12
HPV 31	23 (2.4)	4 (0.8)	19 (4.5)	<0.01	8 (1.3)	1 (0.3)	7 (2.2)	0.06
HPV 33	22 (2.4)	5 (0.9)	17 (3.9)	<0.01	4 (0.6)	1 (0.3)	3 (0.9)	0.62
HPV 35	37 (3.9)	7 (1.3)	30 (7.0)	<0.01	17 (2.7)	3 (1.0)	14 (4.4)	0.01
HPV 39	10 (1.0)	5 (0.9)	5 (1.2)	0.75	2 (0.3)	0 (0.0)	2 (0.6)	0.49
HPV 45	14 (1.5)	5 (0.9)	9 (2.1)	0.17	3 (0.5)	1 (0.3)	2 (0.6)	1.00
HPV 51	17 (1.8)	2 (0.4)	15 (3.5)	<0.01	5 (0.8)	0 (0.0)	5 (1.6)	0.06
HPV 52	54 (5.6)	16 (3.0)	38 (8.9)	<0.01	22 (3.5)	5 (1.6)	17 (5.3)	0.01
HPV 56	17 (1.8)	7 (1.3)	10 (2.3)	0.32	2 (0.3)	0 (0.0)	2 (0.6)	0.49
HPV 58	15 (1.6)	4 (0.8)	11 (2.6)	0.03	4 (0.6)	2 (0.7)	2 (0.6)	1.00
HPV 59	10 (1.0)	0 (0.0)	10 (2.3)	<0.01	1 (0.2)	0 (0.0)	1 (0.3)	1.00

### HIV-Positive Women

Some 44% (427/962) of the women were HIV-positive at baseline. The mean (SD) age of the HIV-positive women was 37 (7) years while their mean (SD) BMI (kilogram per square meter) was 26.9 (5.2).

Any type of HPV infection of the cervix was detected in 44% (186/427) of these women at baseline, while 24% (76/321) had persistence of any type of HPV infections of the uterine cervix. The infection involved a single HPV type at baseline in 23% (100/427) of these women and there was persistence in 19% (62/321). Multiple infections by any type of HPV occurred in 20% (86/427) at baseline and this persisted in 4.4% (14/321) at follow-up (Table 2).

Single lrHPV infections were present in 19% (82/427) and multiple lrHPV infections were present in 6% (27/427) of the women at baseline, while single hrHPV infections were present in 17% (72/427) of the women and multiple hrHPV infections were present in 12% (52/427) of the women at baseline. Single lrHPV infections were persistent in 9% (29/321) and multiple lrHPV infections were persistent in 0.6% (2/321) of the women. Single hrHPV infections were persistent in 13% (42/321) of the women and multiple hrHPV infections were persistent in 3% (9/321) of the women.

The commonest prevalent lrHPV types detected in these women were unspecified HPV types (23/427, 5.4%), HPV66 (23/427, 5.4%), and HPV44 (18/427, 4.2%) while the commonest persistent lrHPV types detected were HPV66 (8/321, 2.5%) and HPV44 (7/321, 2.2%). The commonest prevalent hrHPV types found were HPV52 (38/427, 8.9%) and HPV35 (30/427, 7.0%) while the commonest persistent hrHPV types were HPV52 (17/321, 5.5%) and HPV35 (14/321, 4.4%). HPV16 was detected in only 3% (13/427), while HPV18 was detected in 5% (22/427) of these women at baseline (Table 3). HPV16 and HPV18 were persistent in 1.3% of the HIV-positive women (Table 4).

### Impact of HIV Infection

**Figure 2:**
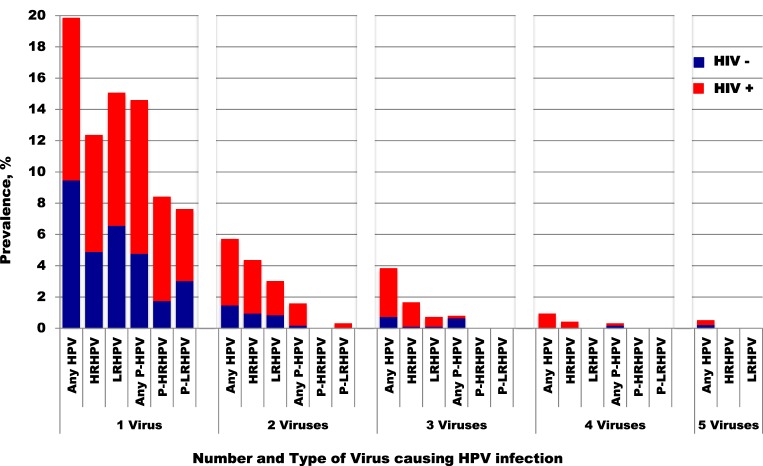
Prevalence of single and multiple HPV infectious by HIV status. HPV, human papillomavirus; HRHPV, high-risk HPV; LRHPV, low-risk HPV; P, persistent. One HIV-positive woman with six any genotypes was excluded from this figure.

**Figure 3:**
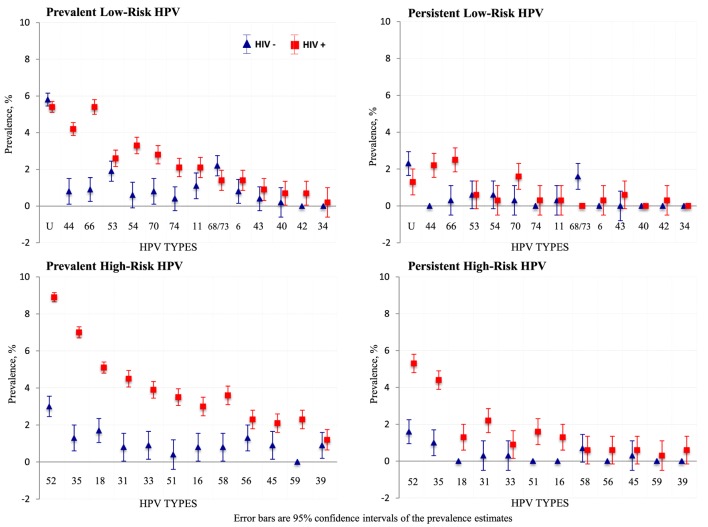
Type-specific prevalence of human papillomavirus (HPV) infections by HIV status. Error bars are 95% confidence intervals of prevalence estimates.

HIV-positive women had higher prevalence, persistence, and presence of multiple HPV infections overall, compared to HIV-negative women (Figure 2). The prevalence of each hrHPV type found in HIV-positive women was also higher than that in HIV-negative women (Figure 3).

In univariate analyses, HIV status and SES were associated with prevalent HPV (Table 5), while HIV status and douching were associated with persistent HPV (Table 6).

**Table 5:** Association between sociodemographic characteristics and potential risk factors for prevalent low-risk and high-risk human papillomavirus (HPV) infections.

Variable	Total *n*	Prevalent low-risk HPV			Prevalent high-risk HPV		
		*n* (%)	Odds ratio (95% CI)	*p*-Value	*n* (%)	Odds ratio (95% CI)	*p*-Value
**Age, years**							
<30	127	29 (23)	1.28 (0.73–2.24)	0.38	27 (21)	1.31 (0.73–2.34)	0.35
30 to <45	659	119 (18)	0.95 (0.62–1.46)	0.83	124 (19)	1.12 (0.72–1.74)	0.59
≥45	176	33 (19)	Ref (1.00)		30 (17)	Ref (1.00)	
**Body mass index, kg/m^2^**							
Normal weight	303	67 (22)	1.38 (0.91–2.09)	0.12	69 (23)	1.73 (1.13–2.65)	0.01
Overweight	324	49 (15)	0.86 (0.56–1.34)	0.52	59 (18)	1.30 (0.84–2.02)	0.22
Obese, ≥30	282	48 (17)	Ref (1.00)		41 (15)	Ref (1.00)	
**Marital status**							
Married	647	114 (18)	0.79 (0.56–1.10)	0.17	115 (18)	0.81 (0.58–1.14)	0.23
Not married	315	67 (21)	Ref (1.00)		66 (21)	Ref (1.00)	
**Education, years completed**							
≤6 years	104	17 (16)	1.11 (0.59–2.07)	0.74	26 (25)	1.50 (0.87–2.60)	0.14
7–12	602	125 (21)	1.48 (1.00–2.21)	0.04	109 (18)	1.00 (0.68–1.46)	1.00
>12	254	38 (15)	Ref (1.00)		46 (18)	Ref (1.00)	
**Socioeconomic status**							
Low	375	82 (22)	1.82 (1.13–2.92)	0.01	87 (23)	1.96 (1.23–3.15)	<0.01
Middle	384	72 (19)	1.50 (0.93–2.42)	0.09	67 (17)	1.37 (0.85–2.23)	0.19
High	203	27 (13)	Ref (1.00)		27 (13)	Ref (1.00)	
**Age at sexual initiation, years**							
≤19	441	88 (20)	1.05 (0.69–1.58)	0.81	86 (21)	1.31 (0.85–2.03)	0.21
19–22	287	46 (16)	0.80 (0.50–1.27)	0.35	59 (21)	1.40 (0.88–2.24)	0.14
>22	219	42 (19)	Ref (1.00)		34 (16)	Ref (1.00)	
**Total lifetime sex partners**							
1	249	45 (18)	1.04 (0.65–1.69)	0.84	34 (14)	0.64 (0.39–1.05)	0.08
2–4	496	97 (20)	1.15 (0.76–1.75)	0.49	104 (21)	1.08 (0.72–1.61)	0.70
≥5	213	37 (17)	Ref (1.00)		42 (20)	Ref (1.00)	
**Douche**							
Yes	606	116 (19)	1.05 (0.75–1.47)	0.75	119 (20)	1.15 (0.82–1.62)	0.40
No	355	65 (18)	Ref (1.00)		62 (17)	Ref (1.00)	
**Vaginal pH**							
<4.5	73	10 (14)	0.69 (0.34–1.38)	0.29	9 (12)	0.61 (0.29–1.26)	0.18
4.5 to <5.5	68	18 (26)	1.57 (0.89–2.77)	0.11	19 (28)	1.69 (0.96–2.95)	0.06
≥5.5	821	153 (19)	Ref (1.00)		153 (19)	Ref (1.00)	
**HIV status**							
Yes	427	109 (26)	2.20 (1.58–3.06)	<0.01	124 (29)	3.43 (2.43–4.86)	<0.01
No	535	72 (13)	Ref (1.00)		57 (11)	Ref (1.00)	

**Table 6:** Association between sociodemographic characteristics and potential risk factors for persistent low-risk and high-risk human papillomavirus (HPV) infections.

Variable	Total *n*	Persistent low-risk HPV			Persistent high-risk HPV		
		*n* (%)	Odds ratio (95% CI)	*p*-Value	*n* (%)	Odds ratio (95% CI)	*p*-Value
**Age, years**							
<30	68	6 (9)	2.36 (0.69–8.04)	0.16	5 (7)	0.83 (0.27–2.51)	0.75
30 to <45	435	39 (9)	2.40 (0.92–6.23)	0.07	47 (11)	1.27 (0.64–2.54)	0.48
≥45	127	5 (4)	Ref (1.00)		11 (9)	Ref (1.00)	
**Body mass index, kg/m^2^**							
Normal weight	195	21 (11)	1.88 (0.88–4.03)	0.10	24 (12)	2.00 (0.96–4.13)	0.06
Overweight	217	12 (5)	0.91 (0.39–2.12)	0.83	23 (11)	1.68 (0.81–3.49)	0.15
Obese, ≥30	183	11 (6)	Ref (1.00)		12 (7)	Ref (1.00)	
**Marital status**							
Married	416	27 (6)	0.57 (0.32–1.03)	0.06	37 (9)	0.70 (0.41–1.20)	0.19
Not married	214	23 (11)	Ref (1.00)		26 (12)	Ref (1.00)	
**Education, years completed**							
≤6 years	74	2 (3)	0.26 (0.05–1.14)	0.07	9 (12)	1.28 (0.53–3.04)	0.57
7–12	390	32 (8)	0.83 (0.44–1.55)	0.55	38 (10)	0.99 (0.54–1.84)	0.99
>12	164	16 (10)	Ref (1.00)		16 (10)	Ref (1.00)	
**Socioeconomic status**							
Low	247	20 (8)	2.11 (0.77–5.77)	0.14	31 (13)	2.09 (0.93–4.71)	0.07
Middle	258	25 (10)	2.57 (0.96–6.89)	0.05	24 (9)	1.50 (0.65–3.44)	0.33
High	125	5 (4)	Ref (1.00)		8 (6)	Ref (1.00)	
**Age at sexual initiation, years**							
≤19	295	30 (10)	2.45 (0.99–6.04)	0.05	33 (11)	1.77 (0.82–3.82)	0.14
19–22	187	13 (7)	1.61 (0.59–4.37)	0.34	21 (11)	1.78 (0.79–4.03)	0.16
>22	136	6 (4)	Ref (1.00)		9 (7)	Ref (1.00)	
**Total lifetime sex partners**							
1	157	13 (8)	1.29 (0.54–3.03)	0.55	8 (5)	0.37 (0.16–0.89)	0.02
2–4	316	27 (9)	1.33 (0.62–2.83)	0.45	36 (11)	0.90 (0.50–1.64)	0.74
≥5	153	10 (7)	Ref (1.00)		19 (12)	Ref (1.00)	
**Douche**							
Yes	407	39 (10)	2.03 (1.01–4.05)	0.04	46 (11)	1.53 (0.85–2.75)	0.14
No	222	11 (5)	Ref (1.00)		17 (8)	Ref (1.00)	
**Vaginal pH**							
<4.5	40	2 (5)	0.58 (0.13–2.51)	0.47	1 (3)	0.23 (0.03–1.77)	0.16
4.5 to <5.5	42	3 (7)	0.86 (0.25–2.89)	0.80	9 (21)	2.54 (1.15–5.61)	0.02
≥5.5	548	45 (8)	Ref (1.00)		53 (10)	Ref (1.00)	
**HIV status**							
Yes	321	31 (10)	1.63 (0.90–2.95)	0.10	51 (16)	4.67 (2.44–8.95)	<0.01
No	309	19 (6)	Ref (1.00)		12 (4)	Ref (1.00)	

**Figure 4:**
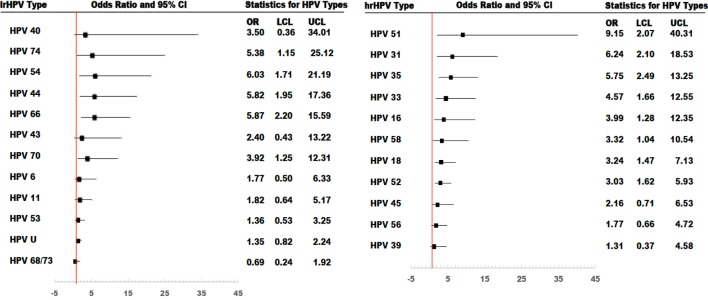
Risk of type-specific prevalent human papillomavirus (HPV) infections among HIV-positive women, compared to HIV-negative women. All models were adjusted. OR, odds ratio; LCL, lower confidenceninterval limit; UCL, upper confidence interval limit.

In age-adjusted analyses comparing HIV-positive and HIV-negative women, the odds ratios (95% CI; *p*-value) were 2.16 (1.55–3.01; <0.001) for prevalent lrHPV; 1.77 (1.23–2.53; 0.001) for prevalent single lrHPV type infection; 3.75 (1.74–8.09; < 0.001) for prevalent multiple lrHPV types; 1.53 (0.84–2.78; 0.16) for low-risk group-specific persistence, and 1.41 (0.77–2.60; 0.25) for persistence of specific lrHPV infections. These associations were relatively unchanged in the multivariate models (Table 7). The associations with HPV 44, 54, 66, 70, and 74 at baseline were significantly stronger in HIV-positive compared to HIV-negative women (Figure 4).

**Table 7:** Risk (odds ratios and 95% confidence interval) of human papillomavirus (HPV) infections by HIV status.

	*n* HIV−/HIV+	Age-adjusted model			Multivariate model		
		HIV-negative	HIV-positive	*p*-Value	HIV-negative	HIV-positive	*p*-Value
**All prevalent HPV infections**							
Any HPV	114/186	1.00	2.81 (2.12–3.73)	<0.001	1.00	2.84 (2.10–3.84)	<0.001
Low risk	72/109	1.00	2.16 (1.55–3.01)	<0.001	1.00	2.09 (1.47–2.97)	<0.001
High risk	57/124	1.00	3.38 (2.39–4.77)	<0.001	1.00	3.38 (2.34–4.87)	<0.001
**Single prevalent HPV**							
Any HPV	91/100	1.00	1.49 (1.08–2.05)	0.013	1.00	1.57 (1.11–2.20)	0.009
Low risk	63/82	1.00	1.77 (1.23–2.53)	0.001	1.00	1.75 (1.20–2.56)	0.003
High risk	47/72	1.00	2.07 (1.40–3.08)	<0.001	1.00	2.12 (1.39–3.21)	<0.001
**Multiple prevalent HPV**							
Any HPV	23/86	1.00	5.47 (3.38–8.85)	<0.001	1.00	5.12 (3.11–8.44)	<0.001
Low risk	9/27	1.00	3.75 (1.74–8.09)	<0.001	1.00	3.42 (1.54–7.56)	0.002
High risk	10/52	1.00	7.15 (3.58–14.3)	<0.001	1.00	6.93 (3.39–14.2)	<0.001
**All persistent HPV infections^a^**							
Any HPV	31/76	1.00	2.68 (1.70–4.22)	<0.001	1.00	2.36 (1.45–3.82)	<0.001
Low risk	19/31	1.00	1.53 (0.84–2.78)	0.16	1.00	1.26 (0.66–2.40)	0.47
High risk	12/51	1.00	4.57 (2.38–8.79)	<0.001	1.00	4.49 (2.26–8.91)	<0.001
**Single persistent HPV^a^**							
Any HPV	30/62	1.00	2.18 (1.36–3.49)	0.001	1.00	1.88 (1.14–3.12)	0.01
Low risk	19/29	1.00	1.41 (0.77–2.60)	0.25	1.00	1.17 (0.61–2.25)	0.62
High risk	11/42	1.00	4.09 (2.05–8.13)	<0.001	1.00	4.13 (2.00–8.55)	<0.001

*Table shows ORs and 95% CI based on logistic regression models; n = number of women*.

*Multivariate models were adjusted for age, marital status, and socioeconomic status*.

*^a^Multivariate models were adjusted for age, marital status, socioeconomic status, age at sexual initiation, and douching*.

For hrHPV infections, the age-adjusted odds ratios (95% CI; *p*-value) comparing HIV-positive women to HIV-negative women were 3.38 (2.39–4.77; <0.001) for prevalent hrHPV; 2.07 (1.40–3.08; <0.001) for prevalent single hrHPV infection; 7.15 (3.58–14.27; <0.001) for prevalent multiple hrHPV infection; 4.57 (2.38–8.79; <0.001) for high-risk group-specific persistence; and 4.09 (2.05–8.13; <0.001) for persistence of single hrHPV infections. These associations were relatively unchanged in multivariate models (Table 7). The effect estimates for HPV 16, 18, 31, 33, 35, 51, 52, and 58 infections at baseline were significantly higher in HIV-positive compared to HIV-negative women (Figure 4).

## Discussion

Our study describes the patterns of prevalent and persistent lrHPV and hrHPV infections among HIV-negative and HIV-positive women in Nigeria, West Africa. HIV-positive women were more likely to have prevalent, persistent, multiple, group-specific, and type-specific lrHPV and hrHPV infections, compared to HIV-negative women.

Little is known about the epidemiology of HPV infections in Africa. Most studies of cervical HPV infections in SSA have been qualitative studies of prevalent hrHPV genotypes because of their well-described association with cervical cancer. However, given that the degree of carcinogenicity of some of the HPV types has not been well elucidated and current studies highlight the marked heterogeneity of HPV infections in this population (8, 22), it is necessary to explore associations between type and group-specific, prevalent and persistent HPV infections, and cervical carcinogenesis in Africa.

Low-risk HPV infections are associated with genital and non-genital warts. Genital warts cause significant physical and psychological morbidities (10, 11). They lack specific treatments, are highly recurrent after treatment, and are associated with significant health-care systems’ costs. The prevalence of lrHPV infections among HIV-negative women in our study is similar to the 18% reported among US women (23) but higher than the prevalence of 4% in South Korean women (24). The prevalence of lrHPV in HIV-positive women in our study (26%) was lower than the 55% reported among HIV-positive women in Rwanda (3).

The commonest lrHPV types in our study were unspecified HPV genotypes, HPV 68/73, and HPV 53 in HIV-negative women while unspecified HPV genotypes, HPV 66 and 44 were the commonest types in HIV-positive women. We did not find any significant associations with group or type specific persistent lrHPV. In a study of peri-urban, black South African women, the commonest lrHPV types were HPV 62 (16%) and 84 (14%) (25).

HPV 34 and 44 were found in only HIV-positive women in this study probably because their prevalence is low in our study population. Interestingly, we found that lrHPV genotypes 44 and 54, which are associated with external anogenital warts (9, 26), and lrHPV genotypes 66 and 70 (9), which are associated with anogenital precancers and cancers, had significantly higher prevalence in HIV-positive women, compared to HIV-negative women. This lends support to the reports of higher prevalence of anogenital warts and cancers, described in previous studies (27–29).

In contrast to our previous study (8), in this enlarged study population, the commonest prevalent hrHPV types among HIV-negative women were HPV 52, HPV 18, and HPV 35 while HPV 52 and 35 were the commonest persistent infections. Other studies found HPV 52 to be the most prevalent hrHPV in SSA (5, 12, 13). HPV 52, 16, and 18 were the most common hrHPV types observed among HIV-negative Ugandan and Kenyan women (6, 7). Among HIV-positive women, the commonest prevalent hrHPV types were HPV 52, 35, and 18, while the commonest persistent hrHPV types were HPV 52, 35, and 31. HPV 52 was also the most prevalent hrHPV genotype observed in a study of HIV-positive Rwandan women (3).

Given the high incidence of cervical cancer in the study population (30), we expected to find more persistent infections among HIV negative and HIV positive woman. Instead, we did not find any HIV-negative woman with persistent HPV 16 or 18 infections while persistent HPV 16 or HPV 18 infections constituted 6.3% of all persistent infections among HIV-positive women. Non-16/18 HPV types may contribute more to cervical cancer development among women in SSA. Many of the cases of persistent hrHPV infections that we observed were with HPV 68/73. Given that HPV 68 is high-risk HPV while HPV 73 is not, studies using technologies that allow differentiation between these two infections should be conducted to examine the association of each HPV type with cervical cancer among women in SSA.

Our findings that the prevalence and persistence of single, multiple, lrHPV and hrHPV infections was higher among HIV-positive compared to HIV-negative women are similar to other reports from SSA (31, 32) The increased prevalence and persistence of HPV infection in immunosuppressed individuals suggests that cell-mediated immune response may play an important role in the resolution and control of HPV infection (33). Although some studies have suggested that the association between HIV infection and increased prevalence of HPV infection and disease is related to the immunosuppression seen in HIV infection, there is also some evidence that mechanisms other than immunosuppression, such as direct molecular interactions between HIV and HPV viral genes, may influence the natural history of HPV (34–37). HIV infection may also increase susceptibility to HPV infections and persistence of infection through alterations of the cytokine response to HPV infection in the cervical mucus (38).

The Advisory Committee on Immunization Practices recommended the 9-valent HPV vaccine (Gardasil 9, Merck and Co., Inc.) as one of three HPV vaccines that can be used for routine vaccination (39). This vaccine protects against HPV 6, 11, 16, 18, 31, 33, 45, 52, and 58, many of which have been observed to be prevalent and persistent in women with single or multiple infections in this study population. Currently, no study has been published on use of the 9-valent HPV vaccine among HIV-positive or negative African women and there are limited data on the response of Africans, regardless of HIV status, to HPV vaccination. The broader coverage provided by the 9-valent vaccine may be sufficient in African populations, regardless of HIV status, and there may be sufficient cross-reactivity to ensure protection of HPV types not included in the vaccine. Additional vaccine development and production of vaccines that focus on the commonest HPV types may be helpful in the African population. Further studies on HPV vaccines, immunologic response of Africans to HPV vaccination, and the roles of non-16/18 hrHPV in cervical carcinogenesis in SSA are warranted.

Our study has several limitations including lack of inclusion of clinical correlates of HIV infection such as duration of HIV infection, use of anti-retroviral therapy, and viral load or CD4 count in the analyses though these factors may be associated with HPV persistence. However, we excluded HIV-positive women who were severely ill from our study.

In conclusion, we found a higher proportion of prevalent and persistent infections with single and multiple lrHPV and hrHPV genotypes in HIV-positive women, compared to HIV-negative women. We also found that non-16/18 hrHPV genotypes were more common in women with persistent infections regardless of HIV status. Our results support the recommendation of the 9-valent HPV vaccine for routine vaccination, especially in SSA populations where non-16/18 hrHPV types are prevalent and likely to cause persistent infections.

## Ethics Statement

The study was conducted according to the Nigerian National Code for Health Research Ethics. Ethical approval to conduct this study was obtained from the Institute of Human Virology Nigeria research ethics committee. Written informed consent was obtained from all participants before enrollment in the study, in accordance with the Declaration of Helsinki.

## Author Contributions

SA conducted the data analyses, interpreted the data, and drafted the manuscript. CA designed and obtained funding for the study. OO and AF processed the samples, performed biochemical assays, and collated the laboratory data. ED contributed to the study coordination. SA, OO, AF, ED, RO, and CA contributed to the study implementation and the manuscript. Each author approved the final version of the manuscript and agreed to be accountable for all aspects of the work.

## Conflict of Interest Statement

The authors declare that the research was conducted in the absence of any commercial or financial relationships that could be construed as a potential conflict of interest.
